# *Cryptococcus bacillisporus* (VGIII) Meningoencephalitis Acquired in Santa Cruz, Bolivia

**DOI:** 10.3390/jof7010055

**Published:** 2021-01-15

**Authors:** Luis Thompson, Lorena Porte, Violeta Díaz, María Cristina Díaz, Sebastián Solar, Pablo Valenzuela, Nicole Norley, Yumai Pires, Fernando Carreño, Sergio Valenzuela, Rukmane Shabani, Volker Rickerts, Thomas Weitzel

**Affiliations:** 1 Unidad de Infectología, Clínica Alemana de Santiago, Facultad de Medicina Clínica Alemana, Universidad del Desarrollo, Santiago 7650568, Chile; lthompson@alemana.cl (L.T.); ssolarp@alemana.cl (S.S.); pablotomas.valenzuela@gmail.com (P.V.); 2 Laboratorio Clínico, Clínica Alemana de Santiago, Facultad de Medicina Clínica Alemana, Universidad del Desarrollo, Santiago 7650568, Chile; lporte@alemana.cl; 3 Servicio de Neurología, Departamento de Neurología y Psiquiatría, Clínica Alemana de Santiago, Facultad de Medicina Clínica Alemana, Universidad del Desarrollo, Santiago 7650568, Chile; vdiaz@alemana.cl; 4 Programa de Microbiologia y Micologia, ICBM, Facultad de Medicina, Universidad de Chile, Santiago 7650568, Chile; mcdiaz@med.uchile.cl; 5 Mycology Section, FG 16, Robert-Koch Institute, 13353 Berlin, Germany; norleyn@rki.de (N.N.); rshabani@berlin-chemie.de (R.S.); 6 Servicio de Anatomía Patológica, Clínica Alemana de Santiago, Facultad de Medicina Clínica Alemana, Universidad del Desarrollo, Santiago 7650568, Chile; ypires@alemana.cl; 7 Departamento de Imágenes, Clínica Alemana de Santiago, Facultad de Medicina Clínica Alemana, Universidad del Desarrollo, Santiago 7650568, Chile; fcarreno@alemana.cl; 8 Servicio de Neurocirugía, Clínica Alemana de Santiago, Facultad de Medicina Clínica Alemana, Universidad del Desarrollo, Santiago 7650568, Chile; valenzuelabasolo@gmail.com; 9 Instituto de Ciencias e Innovación en Medicina (ICIM), Universidad del Desarrollo, Santiago 7550000, Chile

**Keywords:** fungal infections, cryptococcosis, *Cryptococcus gattii* complex, epidemiology, South America

## Abstract

We describe a case of chronic meningoencephalitis with hydrocephalus caused by *Cryptococcus bacillisporus* (VGIII) in an immunocompetent patient from Santa Cruz, Bolivia. This first report of a member of the *Cryptococcus gattii* species complex from Bolivia suggests that *C. bacillisporus* (VGIII) is present in this tropical region of the country and complements our epidemiological and clinical knowledge of this group of emerging fungal pathogens in South America.

## 1. Introduction

Members of the *Cryptococcus gattii* species complex are emerging human and veterinary pathogens found in soils and in association with trees in various parts of the world [1]. Initially, this species complex was considered to inhabit tropical and subtropical regions only, but in recent years it has also been described in temperate latitudes [2,3]. Unlike *C. neoformans*, which mainly causes opportunistic infections, *C. gattii* causes substantial morbidity and mortality in immunocompetent individuals and has the potential to cause outbreaks [1]. Due to recent taxonomical changes of the genus *Cryptococcus*, former *C. gattii* genotypes (VGI–VGIV) were reassigned as five distinct species with differing epidemiological features, with a possible additional genotype/species reported from Sub-Saharan Africa [4,5]. As these nomenclature issues are not yet resolved [4,6,7], in this report, we will use the *C. gattii* species complex nomenclature when referring to all genotypes (VGI–VGV) and use the suggested species names when referring to single genotypes.

In South America, the *C. gattii* species complex is endemic in Brazil and Colombia, although clinical and environmental isolates were also reported from Venezuela, Argentina, Peru, Paraguay, Uruguay, and Chile; most of them belonging to the species *C. deuterogattii* (VGII) and *C. bacillisporus* (VGIII) [8,9]. However, due to the limited availability of molecular diagnostic tools, the exact distribution and clinical relevance of *Cryptococcus* species in South America are incompletely understood [8]. Here, we present the first isolation of *C. gattii* complex in Bolivia, which was from a patient with chronic meningoencephalitis caused by *C. bacillisporus* (VGIII) acquired in the Santa Cruz region.

## 2. Case Report and Results

**Figure 1:**
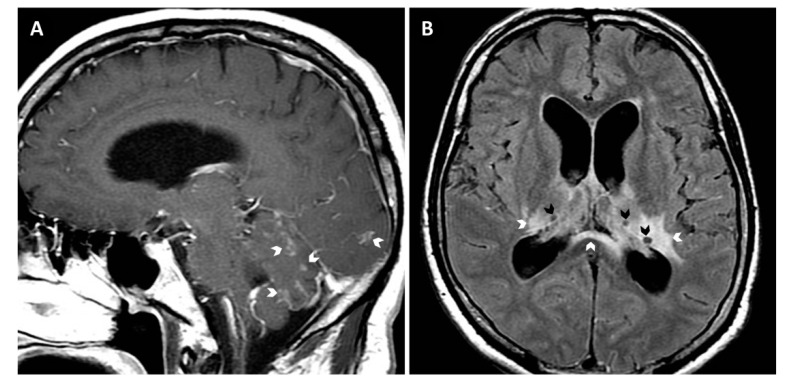
Brain MRI images (**A**, sagittal T1Gd and **B**, axial T2-FLAIR [fluid attenuated inversion recovery] showing generalized leptomeningeal enhancement (arrows in **A**), ganglionar and periventricular edema (white arrows in **B**), cystic lesions suggestive of gelatinous pseudocysts (black arrows in **B**), and enlarged ventricles compatible with communicating hydrocephalus.

A 48-year-old previously healthy man from Santa Cruz, Bolivia, attended Clínica Alemana de Santiago (Chile) with a 12-months history of progressive neurological problems. Symptoms had started with malaise, tremor, and headache, later accompanied by dysphagia, dysphonia, singultus, progressive severe cognitive deterioration, and a weight loss of 10 kg. The patient denied former travel outside Bolivia; his visit to Chile was for medical reasons, i.e., evaluation of the current illness. At presentation, the patient was prostrated and showed severe memory disorder, right central facial paralysis, right brachial and crural paresis, and symmetric postural tremor. Routine laboratory exams were normal, including tests for HIV, syphilis, *Histoplasma capsulatum*, and tuberculosis. While a chest CT scan was unremarkable, brain MRI showed leptomeningeal infiltrates and hydrocephalus, together with ganglionar and periventricular edema and cystic lesions (Figure 1). Cerebrospinal fluid (CSF) examinations revealed low glucose (5 mg/dL), elevated protein (788 mg/dL), and pleocytosis (35 cells/µL, 97% mononuclear); cryptococcal antigen latex agglutination (CALAS^®^, Meridian Bioscience, Memphis, TN, USA) was positive (1:64); Ziehl Neelsen and India ink stains as well as Xpert MTB/RIF were negative. CSF cultures on Sabouraud agar showed small whitish colonies, which produced a blue color on canavanine–glycine–bromothymol blue agar, compatible with *C. gattii* species complex. MALDI-TOF mass spectrometry (VITEK MS, bioMérieux) confirmed the strain as “*C. gattii*” with the maximum confidence score (99.9%). PCR and subsequent restriction fragment length polymorphism (RLFP) analysis of the *URA5* gene classified the strain as *C. bacillisporus* (VGIII). Further multilocus sequence typing (MLST) and specific PCR tests [10,11], performed at the Robert-Koch Institute in Berlin, Germany, grouped the strain as *C. bacillisporus* sequence type 79 (ST79). Antifungal susceptibility testing by microdilution using the Clinical and Laboratory Standards Institute (CLSI) M27 protocol [12] showed the following minimum inhibitory concentrations: amphotericin B (AMB), 0.125 µg/mL; flucytosine, 4 µg/mL; posaconazole, 0.5 µg/mL; voriconazole, 0.25 µg/mL; fluconazole, 32 µg/mL; and itraconazole, 0.25 µg/mL.

**Figure 2:**
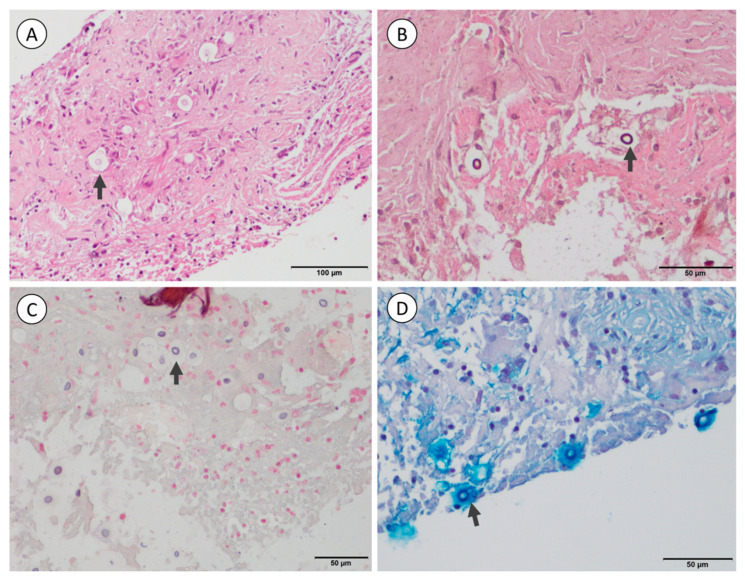
Biopsy of the third ventricle wall showing numerous spherical to oval yeast-like cells (arrows) embedded in a granulomatous reaction with some giant cells. The mucopolysaccharide capsule forms a clear halo around the fungal cells in Hematoxylin and eosin (**A**), Grocott-Gomori’s (**B**), and Fontana-Masson stains (**C**), while with Alcian blue stain (**D**), it is deeply colored.

Monotherapy with liposomal AMB (L-AMB, 3 mg/kg/day) was started since flucytosine is not available in Chile. After 2 weeks and clinical improvement, the patient decided to continue treatment with oral fluconazole (400 mg bid) in Bolivia. Four months later, he returned with severe headache, cognitive impairment, tremor, and severe gait disorder. A brain MRI showed decreased leptomeningeal enhancement, but persistent hydrocephalus and multiple cystic lesions of increased size. CSF samples demonstrated low glucose (11 mg/dL), elevated protein (296 mg/dL), pleocytosis (25 cells/µL, 93% lymphocytes), and were positive for the cryptococcal antigen by latex agglutination (1:64) and lateral flow testing (1:80; CrAg LFA, Immy, Norman, OK, USA). Endoscopic third ventriculocisternostomy was performed, showing cotton-like whitish material (Supplemental Material, Video S1). Biopsies of the ventricle walls revealed granulomatous inflammation with numerous encapsulated yeast-like cells (Figure 2). L-AMB (3 mg/kg/day) was reinitiated in combination with oral fluconazole (400 mg bid). The patient improved clinically and after 2 weeks treatment was switched to consolidation therapy with fluconazole (400 mg bid). Therapy with 400 mg per day was maintained for 2 years. During a follow-up period of 5 years the patient remained clinically stable and without cognitive or functional sequelae, except mild tremor.

**Figure 3:**
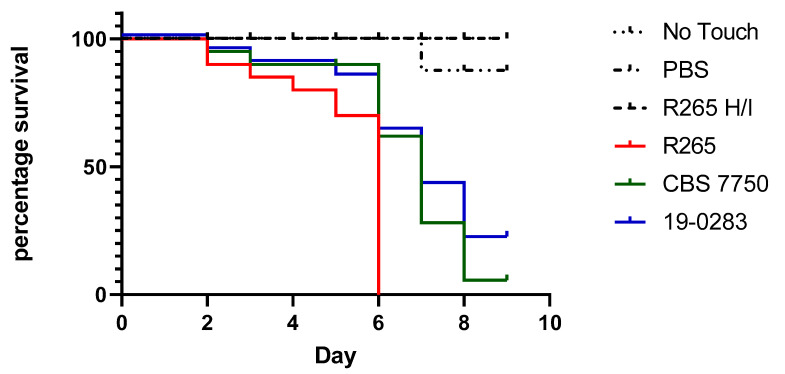
Kaplan–Meyer survival curves of *Galleria mellonella* larvae injected with different *C. gattii* complex isolates and controls (20 larvae per group and experiment). The isolate from Bolivia (19-0283) is represented by the blue line, while *C. gattii* VGII reference strains R265 (high virulence) and CBS 7750 (low virulence) are shown as red and green lines, respectively. Control groups were either non-manipulated larvae (No Touch), or larvae injected with PBS or heat-inactivated (H/I) R265 (black dotted lines). The graph and statistical analysis were performed in Graphpad prism 8.4. The figure represents one of three biological replicates demonstrating equal outcomes.

Due to the protracted clinical presentation of the infection, the virulence of the CSF isolate was evaluated in comparison to previously characterized *C. deuterogattii* (VGII) reference strains. In short, 20 last instar larvae of *Galleria mellonella* weighing 230–330 mg without any grey marks were injected with 10^6^ cryptococci per animal in PBS, as previously described [13]. Two *Cryptococcus* isolates, known to be highly-virulent (R265) and avirulent (CBS7750) in a mouse model [14], were used as comparators. Infected animals were incubated at 37 °C for 9 days and checked daily for survival (movement upon stimulation). Control groups consisted of wax worms, which were (1) not manipulated (no touch), (2) injected with PBS, and (3) injected with heat-inactivated (H/I) R265 (30 min at 65 °C). The experiments revealed a median survival of larvae infected with the Bolivian isolate, CBS7750, and R265 of 7 days, 7 days, and 6 days, respectively. A log–rank test of the survival curve of the Bolivian strain did not differ from CBS7750, but was significantly different to R265 (*p* = 0.0009), suggesting a low virulence of this isolate in this model (Figure 3). 

## 3. Discussion

The reported *C. bacillisporus* case represents the first identification of an isolate of the *C. gattii* species complex in Bolivia. This group of fungal pathogens appears to be of increasing geographical distribution and clinical importance in South America [8]. *C. gattii* complex inhabit >50 tree species in a variety of ecosystems and climates [1]. The exact range and association to ecoclimatic zones in South America is uncertain, but ecological modeling studies from Colombia suggest that the distribution is wide and also includes ecoregions with temperate climate [15]. The Amazon rainforest with its botanical variety has been suggested as the origin of certain strains of higher virulence [1]. Contaminated water sources and infected animals may serve to distribute the fungus in the environment [16]. Unlike *C. neoformans*, some members of the *C. gattii* species complex often affect hosts without known immunocompromise [17]. Risk of disease has been attributed to increased environmental exposure, especially to mature trees, e.g., in the Australian rural-living indigenous population; however, certain dysfunctions of the innate immunity such as anti-granulocyte-macrophage colony-stimulating factor (GM-CSF) autoantibodies might also predispose to central nervous system (CNS) infections with *C. gattii* complex [17].

The taxonomy of human pathogenic *Cryptococcus* species complexes has recently been restructured. The suggested assignation of distinct species instead of genotypes was based on genotypic and phenotypic criteria as well as virulence differences and ecological variations [4]. However, this new taxonomy is still not universally accepted, causing a certain “nomenclatural instability” [6]. The presented patient was infected by *C. bacillisporus*, former *C. gattii* genotype AFLP5, molecular type VGIII. This species is infrequently isolated worldwide, but has been recognized as an emerging pathogen throughout different climate zones in America [18]. Most cases have been described from Colombia, Mexico, southern Brazil, and the West Coast of the USA [1,8,19]. Unlike *C. deuterogattii* (VGII), which causes outbreaks related to few hypervirulent clones, clinical isolates of *C. bacillisporus* (VGIII) have a higher genetic diversity [18]. This species induces lower levels of proinflammatory cytokines than other members of the *C. gattii* complex, leading to prolonged survival and chronic infections [14]. Two main subgroups have been recognized using different techniques; VGIIIa and VGIIIb by multilocus sequence typing [20] and serotypes B and C by restriction fragment length polymorphism (RFLP) analysis of the *CAP59* gene [18]. The presented patient was infected with ST79, which has mainly been isolated in Colombia, but also in Mexico and the USA [18,21,22,23]. ST79 belongs to serotype C, which is more diverse and probably less virulent with less pulmonary infections compared to serotype B [18]. This is in accordance with our results in the *Galleria mellonella* model, suggesting that the Bolivian isolate was of low virulence. As this case suggests, such isolates may still cause CNS infections in non-immunocompromised individuals presenting as subacute meningoencephalitis without evident signs of infection such as fever [24], in contrast to the typical presentations of *C. deuterogattii* (VGII), often involving pulmonary manifestations. The chronic course of *C. bacillisporus* (VGIII) together with the prolonged incubation period may lead to misdiagnosis or significant delays including the risk of a fatal outcome [1,25,26]. The under-recognition of this pathogen is also related to diagnostic difficulties, since the necessary cultural and molecular techniques are mostly unavailable in low- and middle-income countries [8]. In Bolivia, for example, all reported cryptococcosis cases up to now have been diagnosed using India ink, without species differentiation [8,27,28]. An additional problem is that commercial tests may not adequately identify cryptococcosis caused by *C. gattii* [29]. 

The clinical management in resource-poor settings is problematic, as essential antifungals are often not accessible or of uncertain quality [30]. Flucytosine, for example, is still unavailable in most South American countries [31], although the drug is designated as a core medicine in the WHO Model List of Essential Medicines since 2015 [32]. A further therapeutic challenge is the management of increased intracranial pressure, which might have an atypical presentation (“frozen state”) [33], and often requires neurosurgical intervention [34].

In conclusion, our report suggests that *C. bacillisporus* (VGIII) is present in the tropical lowlands east of the Andes Mountains in Bolivia and has to be considered as a cause of CNS infections in this region. Further studies are necessary to understand the distribution and clinical relevance of this and other *C. gattii* complex species in Bolivia and neighboring countries. Our case also highlights the importance of diagnostic cooperation with mycology reference laboratories across national borders, to improve individual patient management and also to understand the epidemiological landscape and risk factors regarding this group of emerging fungal pathogens.

## Supplementary Materials

The following are available online at https://www.mdpi.com/2309-608X/7/1/55/s1. Video S1: Endoscopic third ventriculocisternostomy showing white cotton-like material floating in CSF and attached to the ventricle walls.
